# Analytical and clinical validation of a NGS panel in detecting targetable variants from ctDNA of metastatic NSCLC patients

**DOI:** 10.1002/cam4.70078

**Published:** 2024-10-09

**Authors:** Feifei Fan, Guozhong Jiang, Juan Lv, Hongmin Wang, Wenjie Li, Chenglin Liu, Yu Zhao, Zhou Zhang, Haiwei Du, Zhihong Zhang, Xiangnan Li, Wen‐cai Li

**Affiliations:** ^1^ Department of Respiratory Medicine The First Affiliated Hospital of Zhengzhou University Zhengzhou China; ^2^ Department of Pathology The First Affiliated Hospital of Zhengzhou University Zhengzhou China; ^3^ Burning Rock Biotech Guangzhou China; ^4^ Department of Thoracic Surgery and Lung Transplantation The First Affiliated Hospital of Zhengzhou University Zhengzhou China

**Keywords:** circulating tumor DNA, concordance, next‐generation sequencing, targetable variants, tissue sample

## Abstract

**Background:**

Circulating tumor DNA (ctDNA) has emerged as a promising biomarker for noninvasive cancer diagnostics, particularly in the context of metastatic non‐small‐cell lung cancer (NSCLC). Detecting targetable variants through ctDNA analysis offers the potential to guide treatment decisions, especially in cases where tissue samples are insufficient or unavailable.

**Method:**

In this study, we developed and validated a next‐generation sequencing panel targeting 101 cancer‐related genes (101‐test) to detect somatic variants in ctDNA from a large cohort of Chinese patients with metastatic NSCLC. The performance of the 101‐test was assessed by evaluating its limit of detection (LOD), accuracy, and precision in identifying molecular variants. Additionally, the concordance between ctDNA and tissue samples for detecting targetable variants was analyzed in 904 patients.

**Results:**

The 101‐test demonstrated a LOD of 0.38% for single‐nucleotide variants (SNVs), 0.33% for insertions and deletions (InDels), and 0.33% for fusions. Sensitivity was 98.3% for SNVs, 100% for InDels, and 100% for fusions when compared to digital droplet PCR (ddPCR)/breakpoint PCR reference methods. The by‐variant sensitivity for somatic variants was 97.5%, with a specificity of 99.9% between tumor‐only and tumor‐normal analyses. In a real‐world cohort, the concordance between ctDNA and tissue samples for identifying targetable variants was 72.2% (457/633). Notably, the EGFR S768I variant, recently recommended by clinical guidelines, achieved an 80% concordance rate. Furthermore, 4.3% of patients (27/633) with targetable variants were identified exclusively through ctDNA testing.

**Conclusion:**

The ctDNA‐based 101‐test is a highly sensitive and specific tool for detecting targetable variants in metastatic NSCLC, particularly in cases with insufficient tissue samples. The findings support the use of ctDNA testing as a reliable and complementary method to traditional tissue‐based molecular analysis, enhancing the precision of treatment strategies for NSCLC patients.

## Graphical abstract

The detection of targetable variants from circulating tumor DNA (ctDNA) has the potential to serve as a surrogate for tissue‐based molecular testing. In this study, we developed a next‐generation sequencing panel consisting of 101 cancer‐related genes (101‐test) and evaluated its performance in detecting somatic variants from ctDNA in a large cohort of Chinese patients with metastatic non‐small cell lung cancer.

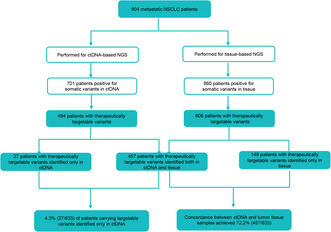

## 1 INTRODUCTION

Lung cancer is the leading cause of cancer‐related death worldwide and in China.^1^, ^2^ Non‐small cell lung cancer (NSCLC) accounts for approximately 85% of all lung cancers.^1^ It is well known that oncogenic driver variants are responsible for 30% of NSCLCs in the Western population and 60% in the Chinese population.^3^, ^4^ Targeted therapies against oncogenic drivers, such as epidermal growth factor receptor (*EGFR*), anaplastic lymphoma kinase (*ALK*), ROS proto‐oncogene 1 (*ROS1*), mesenchymal‐epithelial transition (*MET*), rearranged during transfection (*RET*), and neurotrophic tyrosine receptor kinase (*NTRK*), have become the standard of care for patients with advanced or metastatic NSCLC.^5^, ^6^

Molecular testing of tumor tissues is the preferred method for identifying these oncogenic drivers,^7^, ^8^ but up to 30% of patients with NSCLC have insufficient tumor tissue specimens for molecular testing.^9^, ^10^ In recent years, circulating tumor DNA (ctDNA), derived from plasma cell‐free DNA (cfDNA), has become an increasingly alternative method for detecting molecular variants in patients with NSCLC without sufficient tumor tissues.^11^, ^12^, ^13^

Next‐generation sequencing (NGS) technology has revolutionized cancer molecular diagnostics owing to its ability to simultaneously assess mutations, amplifications, deletions, fusions, microsatellite instability status, and tumor mutational burden.^14^, ^15^ Several liquid biopsy NGS companion diagnostic tests have become commercially available.^16^, ^17^, ^18^, ^19^ However, detecting variants with an allele frequency (AF) below 0.5% from ctDNA remains a challenge for DNA sequencing assays.^19^ A recent study documented that the 101‐test has high sensitivity in detecting variants below 0.5% AF from ctDNA.^19^

In this study, we disclosed the analytical and clinical performance of the 101‐test in detecting targetable ctDNA variants in a large real‐world cohort of Chinese patients with metastatic NSCLC.

## 2 MATERIALS AND METHODS

### 2.1 Healthy donors

A total of 336 healthy donors who underwent physical examination at The First Affiliated Hospital of Zhengzhou University were enrolled. Ten milliliters of peripheral blood were collected from each healthy donor with informed consent obtained for the use of their peripheral blood.

### 2.2 Patient selection

NSCLC patients who met the following inclusion criteria were enrolled from The First Affiliated Hospital of Zhengzhou University between December 2014 and January 2019^1^: above18 years old^2^; had metastatic disease (Stage IV)^3^; underwent tissue biopsy to direct future treatment; and^4^ both tumor tissue and peripheral blood samples were collected. Patients underwent tumor staging according to the 8th edition of the Tumor, Node, Metastasis (TNM) classification of NSCLC. This study was approved by the Ethics Committee of the First Affiliated Hospital of Zhengzhou University (2019‐KY‐30), and informed consent was obtained from each patient for the use of peripheral blood and tumor tissue samples.

### 2.3 DNA extraction

To extract DNA, 10 mL of whole blood was collected and centrifuged at 2000*g* for 10 min at 4°C. The supernatant was transferred to a 15 mL centrifuge tube and centrifuged at 16,000 × ***g*** at 4°C for 10 min. The extracted cfDNA was obtained from the plasma using the QIAamp Circulating Nucleic Acid Kit (Qiagen, Valencia, CA, USA) following the manufacturer's instructions. Genomic DNA was extracted from WBC and cell lines using the MagPure Universal DNA Kit (Magen, Guangzhou, China), according to the manufacturer's instructions. Tissue DNA was extracted using a QIAamp DNA formalin‐fixed paraffin‐embedded (FFPE) tissue kit (Qiagen, Valencia, CA, USA), following the manufacturer's instructions. The concentration of tissue DNA, cfDNA, and genomic DNA isolated from cell lines was measured using a Qubit Fluorometer with a Qubit double‐stranded DNA assay kit (Life Technologies, Carlsbad, CA, USA).

### 2.4 DNA library construction

To construct the DNA library, a total of 20–80 ng of cfDNA was end‐repaired, phosphorylated, dA‐tailed, and adaptor ligated for library construction. The DNA library was purified using SPRI beads.

### 2.5 Capture‐based targeted sequencing

Capture‐based targeted sequencing was performed on samples from a Clinical Laboratory Improvement Amendments (CLIA)‐certified College of American Pathology (CAP)‐accredited laboratory using a panel consisting of 101 cancer‐related genes in the human genome (Burning Rock Biotech, Guangzhou, China). The panel included the whole exons or selected exons of 99 genes and introns of 10 genes. The gene list for the 101‐test is summarized in Table S1. Libraries were sequenced on an Illumina NextSeq 500/550/550Dx (Illumina, Inc., San Diego, CA, USA) or NovaSeq 6000 (Illumina, Inc., San Diego, CA, USA) with paired‐end reads. The mean coverage depths for the targeted sequencing of tissues and cfDNA samples were 1722× and 15,880×, respectively (Figure S1).

### 2.6 Sequencing data analysis

Sequencing data were processed using a customized bioinformatics pipeline designed to detect single‐nucleotide variants (SNV), small insertions and deletions (InDels), copy number variants (CNV), and fusions. Raw sequencing data were preprocessed using bcl2fastq, and preprocessed sequencing data were mapped to the human genome (hg19) using Burrows‐Wheeler Aligner 0.7.10 to generate BAM files. Variant calling was performed using Vardict to detect SNVs/InDels. False‐positive SNVs/InDels identified in cfDNA from healthy donors were used to filter the background variants. The variants were annotated using ANNOVAR and SnpEff v3.6.

The detected variants were grouped into four categories according to their level of clinical significance, as defined in the Association for Molecular Pathology, American Society of Clinical Oncology, and College of American Pathologists guidelines.^20^ Category I comprised variants with strong clinical significance with supporting evidence of FDA‐approved treatments, recommendations from professional guidelines, or well‐powered studies with consensus from field experts.

### 2.7 Cell lines and serial dilution experiments

To evaluate the performance of the NGS‐based 101‐test, genomic DNA was extracted from 33 cell lines (Table S2). Two pooled DNA samples were created by mixing the genomic DNA from 19 (pooled DNA sample 1) and 14 (pooled DNA sample 2) cell lines in equal proportions. NA12878, a human DNA standard, was used to dilute the pooled DNA samples. Molecular variants were identified and their AFs were calculated. The variants were categorized into four groups based on their AFs.

### 2.8 Statistical analysis

Accuracy, precision, and 95% limit of detection (LoD95, lowest concentration at which 95% of positive samples were detected) in identifying somatic variants were evaluated in silico simulated samples, cfDNA reference standards (ctDNA v2 Reference Materials, SeraCare, Milford, MA, USA), and in vitro serial dilution cell lines. The chi‐squared test was used to compare differences in categorical variables between the two groups. The sensitivity (positive predictive agreement, PPA), specificity (negative predictive agreement, NPA), positive predictive value (PPV), and positive concordance rate were calculated using tissue biopsy samples as references. The positive concordance rate was defined as the ratio of the number of patients with positive variants in both tissue and ctDNA to the number of patients with positive variants in either tissue or ctDNA. All statistical analyses were performed using R 3.3.3 (https://www.r‐project.org/). A two‐sided *p*‐value of 0.05 was set as the level of significance.

## 3 RESULTS

### 3.1 Determination of limit of blank

The limit of blank (LoB) of the 101‐test was determined by sequencing cfDNA from 120 healthy donors. Our study observed a zero false positive rate for panel‐wide fusions and CNVs and a near‐zero false positive rate for panel‐wide SNVs/InDels with a frequency less than 0.00003% (Table 1). Additionally, our study observed a zero false‐positive rate for Category I variants (Table 1).

**TABLE 1:** Summary results of LoB study.

Variants	Per position false positive rate	Per sample false positive rate
Category I variants	0%	0% (0/120)
Panel‐wide SNVs/InDels	<0.00003% (6/174,966 × 120)	5% (6/120)
Panel‐wide Fusions	0%	0% (0/120)
Panel‐wide CNVs	0%	0% (0/120)

Abbreviations: CNV, copy number variant; Indel, insertion and deletion; LoB, limit of blank; SNV, single‐nucleotide variant.

### 3.2 In silico simulation experiments to determine the limit of detection (LoD) for identifying molecular variants

To determine the sensitivity of the 101‐test for detecting SNVs/InDels/fusions, capture‐based targeted sequencing was performed on 20 cfDNA samples derived from healthy donors. BAM files of these 20 plasma samples were utilized to model 122 SNVs/InDels, including 49 hotspots and 73 non‐hotspot SNVs/InDels (Table S3) over a wide range of genomic loci in 41 genes at defined allele frequencies (AF) of 0.1%, 0.3%, 0.5%, 0.7%, and 1%, and model fusions occurring in 9 genes, including *ALK*, *ROS1*, *RET*, *FGFR1*, *FGFR2*, *FGFR3*, *NTRK1*, *NTRK3*, and *NRG1* (Table S3) at defined AF of 0.2%, 0.4%, 0.6%, 0.8%, and 1%, respectively. The artificial BAM files were subsequently randomly sampled with median sequencing depths of 8000×, 10,000×, and 15,000×. In total, 600 simulated samples were generated. SNVs/InDels/fusions were also identified. In silico simulation experiments demonstrated that the 101‐test accurately detected hotspot SNVs/InDels with an AF of 0.3% and non‐hotspot SNVs/InDels with an AF of 0.7% (Table S4). Moreover, fusions with an AF as low as 0.2% could be accurately detected with 94.63% sensitivity and 100% specificity (Table S4).

BAM files of 216 cfDNA samples from healthy donors were used to model CNVs occurring in 13 genes, including *FGFR2*, *CCND1*, *KRAS*, *CDK4*, *ERBB2*, *YES1*, *ALK*, *FGFR3*, *EGFR*, *CDK6*, *MET*, *FGFR1*, and *MYC*, with copy number gains of 2.1, 2.3, 2.5, and 2.7, respectively. CNV occurring in each gene were simulated 20 times. Finally, 1040 simulated samples were generated. In silico simulation experiments indicated that the 101‐test could detect CNVs with a copy number gain of 2.3 with 92.69% sensitivity and 100% specificity (Table S4).

Collectively, our in silico data indicate that the 101‐test is reliable for detecting hotspot SNVs/InDels with an AF≥0.3%, non‐hotspot SNVs/InDels with an AF ≥0.7%, fusions with an AF ≥0.2%, and CNVs with a copy number gain ≥2.3.

### 3.3 In vitro experiments to determine the LoD of the 101‐test in detecting genomic variants

**FIGURE 1:**
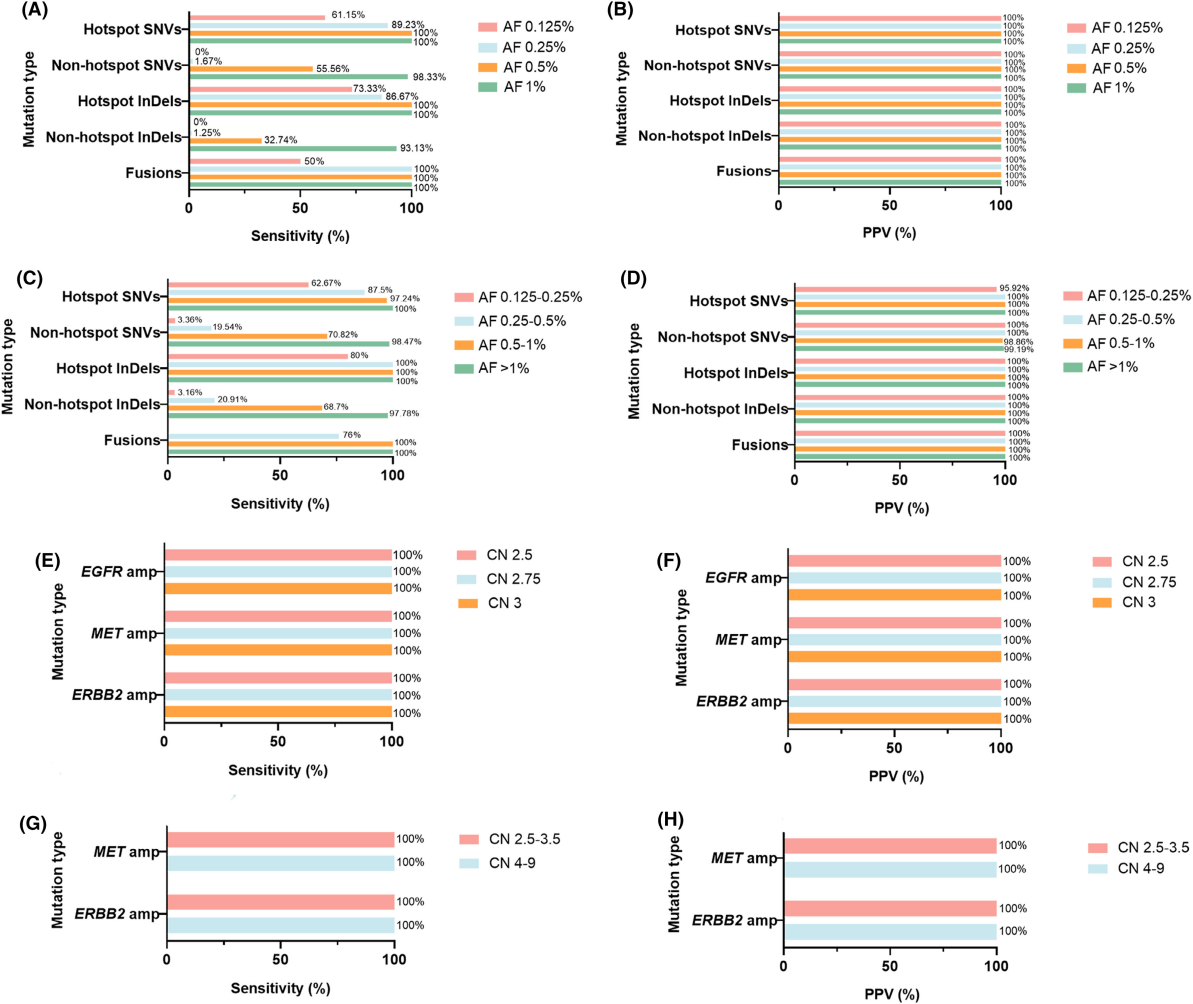
Analytical validation of the 101‐test in detecting SNVs, InDels, and fusions using commercial cfDNA reference standards and pools of genomic DNA from human tumor cell lines. The sensitivity (A) and PPV (B) of the 101‐test in detecting SNVs, InDels, and fusions at different allele frequencies using commercial cfDNA reference standard; the sensitivity (C) and PPV (D) of the 101‐test in detecting SNVs, InDels, and fusions at different allele frequencies using pools of genomic DNA from human tumor cell lines; the sensitivity (E) and PPV (F) of the 101‐test in detecting CNVs with different copy number from human tumor cell lines; the sensitivity (G) and PPV (H) of the 101‐test in detecting CNVs with different copy number from human plasma samples. cfDNA, cell‐free DNA; SNV, single‐nucleotide variant; InDel, insertion and deletion; CNV, copy number variant; PPV, positive predictive value; AF, allele frequency; CN, copy number; amp, amplification.

In order To determine the LoD of the 101‐test in detecting SNVs/InDels/fusions, in vitro experiments were performed using commercial cfDNA reference standards carrying 30 SNVs/InDels in 20 genes and two fusions in two genes (Table S5). The AF of the cfDNA reference standards were 0.125, 0.25, 0.5, and 1%. Capture‐based targeted sequencing was performed 20 times on each cfDNA reference sample. The results showed that the sensitivity of hotspot SNVs with an AF of 0.25% and 0.5% was 89.23% and 100%, non‐hotspot SNVs with an AF≥1% was 98.33%, hotspot InDels with an AF of 0.25% and 0.5% were 86.67% and 100%, non‐hotspot InDels with an AF≥1.0% was 93.13%, and fusions with an AF of 0.25% were 100% (Figure 1A). Moreover, the PPV of the abovementioned variants was 100% (Figure 1B).

Next, we explored the LoD of the 101‐test in detecting SNVs/InDels/fusions in the pools of cancer cell lines, in vitro experiments were performed with human genome reference standard DNA (NA12878) and DNA isolated from human tumor cell lines carrying somatic variants, including SNVs, InDels, and fusions, confirmed by externally capture‐based targeted sequencing using a large panel consisting of 520 cancer‐related genes (OncoScreenPlus, Burning Rock Biotech, Guangzhou, China).^21^ In total, 33 cancer cell lines carried 208 SNVs, 25 InDels, and 5 fusions in 64 genes (Table S3). Variants with AF≥10%, confirmed by the OncoScreenPlus assay, were used for further analyses. Our analyses demonstrated that the sensitivity of hotspot SNVs with an AF of 0.5%–1%, non‐hotspot SNVs with an AF≥1.0%, hotspot InDels with an AF of 0.25%–0.5%, non‐hotspot InDels with an AF≥1%, and fusions with an AF of 0.5%–1% were 97.24% (PPV = 100%), 98.47% (PPV = 99.19%), 100.0% (PPV = 100%), 97.78% (PPV = 100%), and 100.0% (PPV = 100%) (Figure 1C,D). Next, the LoD was determined using the conservative hit‐rate approach for SNVs/InDels/fusions. LoD by hit rate was defined as the mean AF value at the lowest dilution level tested, with at least 95% detection across replicates. The estimated LoDs for hotspot SNVs, non‐hotspot SNVs, hotspot InDels, non‐hotspot InDels, and fusions were 0.38%, 1%, 0.33%, 1%, and 0.33%, respectively.

To assess the LoD of the 101‐test for detecting CNVs, we tested its performance on DNA extracted from three different cell lines: MDA‐MB‐468 with *EGFR* amplification (CN = 45), Hs746T with *MET* amplification (CN = 17), and HCC1954 with *ERBB2* amplification (CN = 57). To generate CNVs with the expected CN of 2.5, 2.75, and 3, we diluted the extracted DNA with NA12878. DNA samples were tested using the 101‐test to evaluate their ability to detect CNVs with different CN. The results showed that the sensitivity and positive predictive value (PPV) of the 101‐test were both 100% for CNVs with a CN of 2.5, 2.75, and 3 (Figure 1E,F), indicating that the LoD of the test for detecting CNVs was a CN of 2.5. We then evaluated the performance of the 101‐test in detecting CNVs in plasma samples from patients with tumors with CNVs (*n* = 24, confirmed by ddPCR) and healthy donors (*n* = 120). The 101‐test showed a sensitivity of 100% (with a PPV of 100%) in detecting CNVs with a CN of 2.4–3.5 and 4–9 in the plasma samples (Figure 1G,H). Furthermore, CNVs from healthy donors was 100% negative by the 101‐test.

Taken together, the results from the in vitro experiments indicate the reliability of the 101‐test in detecting molecular variants at the LoD.

### 3.4 Precision studies by in vitro experiments

**FIGURE 2:**
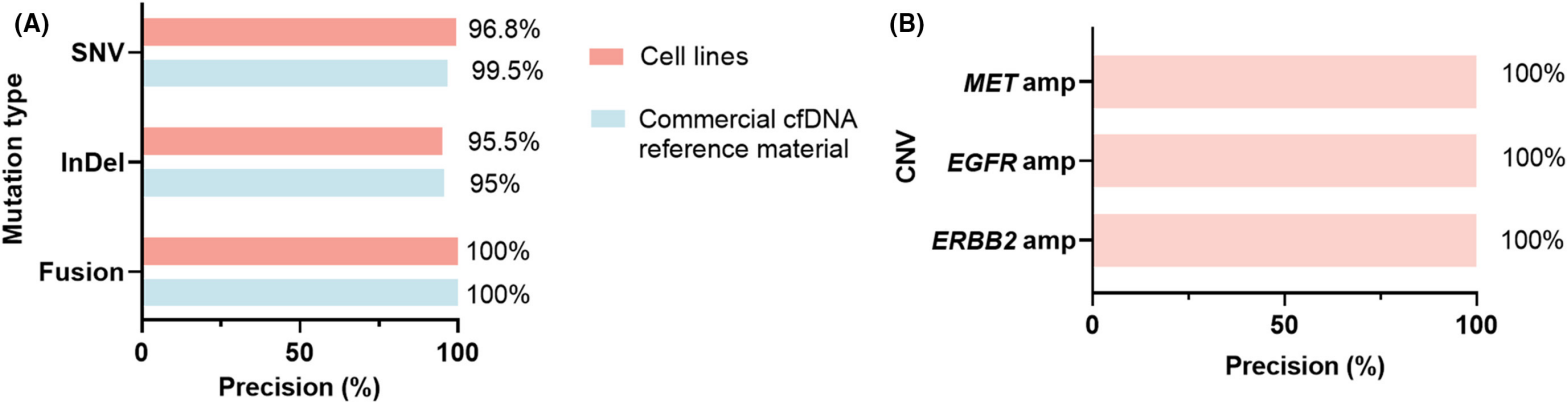
Analytical precision of the 101‐test in detecting somatic variants. (A) The precision of the 101‐test in detecting SNVs, InDels, and fusions in commercial cfDNA reference materials and pools of genomic DNA from cell lines carrying SNVs/InDels/fusions; (B) the precision of the 101‐test in detecting CNVs in cell lines carrying CNVs. amp, amplification; cfDNA, cell‐free DNA; CNV, copy number variant; InDel, Insertion and deletion; PPV, positive predictive value; SNV, single‐nucleotide variant.

To further assess the analytical precision of the 101‐test in the detection of somatic variants, in vitro experiments were performed using commercial cfDNA reference and human cell lines. DNA samples for NGS were prepared by three operators using different sets of reagents, with three technical replicates each (eight technical replicates for commercial cfDNA reference with an AF of 1.0%) on 3 different days. Variants with an AF of 1% in the commercial cfDNA reference standard, hotspot variants with an AF ranging from 0.4% to 0.8%, and non‐hotspot variants with an AF ranging from 1.0% to 2.0% identified in pooled cell line DNA samples were used to assess the analytical precision of the 101‐test in detecting somatic SNVs/InDels/fusions. Diluted cell lines carrying a copy number gain ranging from 2.4 to 3.3 in *MET*, *ERBB2*, and *EGFR* were used to assess the analytical precision of the 101‐test in detecting CNVs. For the commercial cfDNA reference, the precision of the panel‐wide SNVs, InDels, and fusions was 99.5%, 95%, and 100%, respectively. For pooled cell line DNA samples, the precision of panel‐wide SNVs, InDels, and fusions was 96.8%, 95.5%, and 100%, respectively (Figure 2A). CNVs achieved 100% precision (Figure 2B).

The performance of the 101‐test in detecting somatic variants from ctDNA without paired WBCs.

In routine clinical practice, WBC/non‐cancerous samples are commonly used to filter out germline variants to identify somatic variants in plasma samples (conventional method). However, NGS of WBC/non‐cancerous samples introduces a long turnaround time and high cost. In this study, whether the 101‐test could accurately predict alterations by plasma‐only sequencing. Plasma and matched WBCs were collected from 100 patients with human malignancies for 101 tests. We found that the by‐variant sensitivity of somatic tumor variants was 97.5% with a specificity of 99.93% when the conventional method was used as a reference (Table 2).

**TABLE 2:** Comparison of the performance of the 101‐test to conventional test in detecting molecular variants.

101‐test	Conventional test		
	Somatic variants	Germline variants	
Somatic variants	153	4	PPV = 97.5%
Germline variants	4	6035	NPV = 99.93%
	Sensitivity = 97.5%	Specificity = 99.93%	Accuracy = 99.87%

Abbreviations: NPV, negative predictive value; PPV, positive predictive value.

Clonal hematopoiesis (CH), which results from the accumulation of somatic variants in hematopoietic stem cells, can also be detected by ctDNA sequencing. In the present study, only 0.11 nontumor somatic variants per ctDNA sample (11/100) were identified as somatic tumor variants (Table 3). Taken together, the 101‐test is a feasible and reliable tool for identifying somatic tumor variants from ctDNA without paired WBC samples.

**TABLE 3:** Eleven variants derived from clonal hematopoiesis in ctDNA sample.

Gene	Alteration	Alteration type	AF in WBC samples	AF in cfDNA sample	Clinical significance
PDGFRA	p.K812N	Missense	0.1%	0.50%	Unknown
STK11	p.R86G	Missense	1.02%	1.11%	Unknown
ARID1A	p.H1278P	Missense	0.53%	0.67%	Unknown
NFE2L2	p.L74V	Missense	0.68%	0.60%	Unknown
APC	p.S1465fs	Frameshift	0.16%	1.6%	Potential
PIK3R1	p.F398fs	Frameshift	0.3%	0.57%	Potential
CHEK2	c.1096‐1G > A	Splice site	0.28%	0.86%	Potential
POLE	p.N563S	Missense	1.28%	0.90%	Unknown
TP53	p.R273C	Missense	0.7%	0.84%	Potential
MLH1	c.1039‐8_1039‐7insTTA	Splice site	0.25%	1.91%	Unknown
KEAP1	p.G350S	Missense	0.72%	0.54%	Unknown

Abbreviations: AF, allele frequency; ctDNA, circulating tumor DNA; WBC, whole blood cell.

### 3.5 Clinical validation of concordance of molecular variants between the 101‐test and conventional method(s)

**FIGURE 3:**
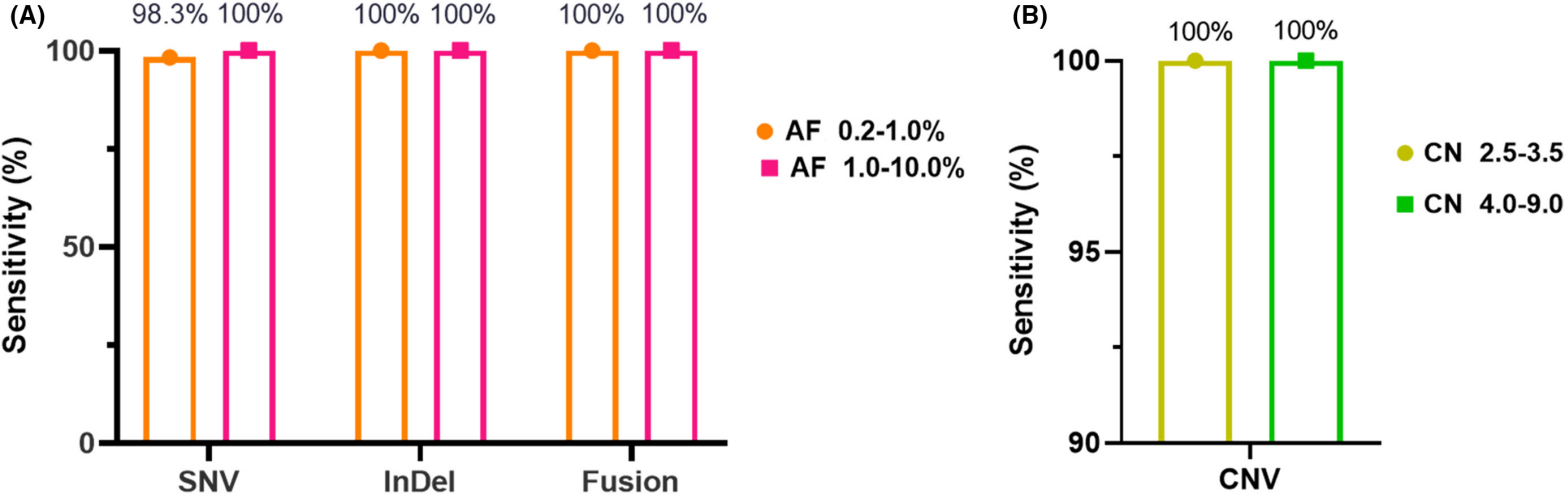
Clinical validation of concordance of molecular variants between the 101‐test and conventical method(s). (A) The sensitivity of the 101‐test in detecting SNVs, InDels, and fusions at different allele frequencies; (B) the sensitivity of the 101‐test in detecting CNVs at different copy numbers. AF, allele frequency; CN, copy number; CNV, Copy number variant; InDel, Insertion and deletion; SNV, Single‐nucleotide variant.

To assess the accuracy of the 101‐test in predicting SNVs/InDels/fusions and CNVs from plasma samples, 185 plasma samples from patients with human malignancies were obtained, including 161 samples with at least one actionable driver SNV(s)/InDel(s)/fusion(s) confirmed by ddPCR or breakpoint PCR and 24 samples with at least one actionable driver CNV(s) confirmed by ddPCR. The sensitivity of the 101‐test in predicting SNVs with an AF of 0.2%–1%, Indels with an AF of 0.2%–1%, and fusions with an AF of 0.2%–1.0% were 98.3%, 100.0%, and 100.0%, respectively, when the conventional method(s) was used as a reference (Figure 3A). The sensitivity of CNVs with a copy number gain of 2.5–3.5 was 100% (Figure 3B) when ddPCR was used as a reference.

### 3.6 Clinical validation of the 101‐test in detecting targetable variants from ctDNA of patients with metastatic NSCLC

Next, the clinical performance of somatic variant detection from paired tissue and blood samples using the 101‐test was investigated in a large cohort of 904 patients with stage IV NSCLC. Clinical characteristics are summarized in Table S6. The interval between tissue and plasma sampling ranged from 0 to 10 days.

The distribution of genomic alterations in the paired tissue and plasma samples is shown in Figure S2. The somatic variant detection rates for the tissue and plasma samples reached 95.1% (860/904) and 77.5% (701/904), respectively. In addition, the detection rates of targetable variants in the tissue and plasma samples were 67.04% (606/904) and 53.54% (484/904), respectively (Table S7). *EGFR* mutation, *ALK* fusion, *MET* amplification, *MET* exon 14 skipping mutation, *ERBB2* amplification, *BRAF* V600E, *ROS1* fusion, *RET* fusion, and *NTRK* fusion were detected in 51.44%, 6.53%, 7.52%, 0.66%, 4.2%, 0.88%, 1.55%, 1.44%, and 0.11% of patients, respectively (Table S7).

**FIGURE 4:**
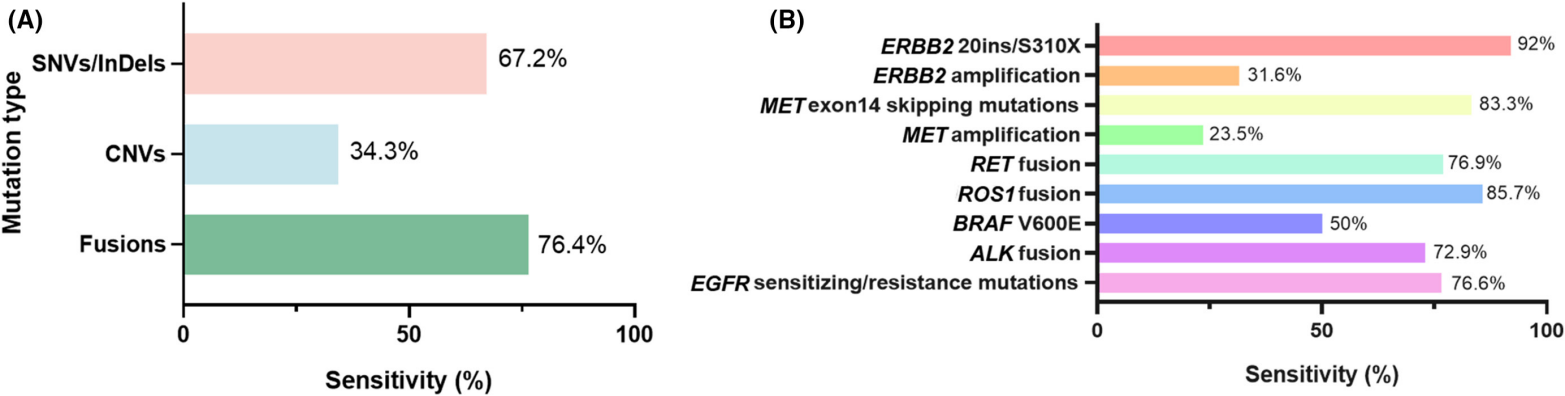
Clinical validation of the 101‐test in detecting targetable variants from ctDNA of patients with metastatic NSCLC. (A) The sensitivity of the 101‐test in detecting SNV/InDels, CNVs, and Fusions in plasma, when tumor‐based NGS was used as a reference; (B) the sensitivity of the 101‐test in detecting a certain actionable alteration in plasma, when tumor‐based NGS was used as a reference. CNV, copy number variant; ctDNA, circulating tumor DNA; InDel, insertion and deletion; SNV, single‐nucleotide variant.

The concordance of somatic variants identified between tissue‐based and plasma‐based NGS was calculated either by variant or patient. The by‐variant sensitivity of plasma testing was 67.2% (with a PPV of 82.4%), 76.4% (PPV of 93.1%) for SNVs/InDels and fusions, and 34.4% for CNVs with a PPV of 59.6% (Figure 4A, Table S8). The by‐variant sensitivities of *ROS1* (85.7%), *MET* ex14 skipping mutation (83.3%), and *EGFR* sensitizing/resistance mutation (76.6%) were more than 75% (Figure 4B). Similar results were observed in patient concordance analyses. We found that the by‐patient sensitivities of plasma testing for predicting targetable SNVs, InDels, fusions, and CNVs were 75.55%, 78.7%, 73.08%, and 26.42%, respectively, when paired tissue was used as a reference. Furthermore, patient sensitivity was the highest for *ROS1* fusion (87.5%), followed by *MET* ex14 skipping mutation (83.33%), *EGFR* S768I (80.0%), *EGFR* 19del (78.95%), *EGFR* L858R (77.47%), *KRAS* G12X (77.94%), *EGFR* T790M (73.91%), and *ALK* fusion (71.23%) (Table 4). Among the 858 targetable variants identified in tissue or plasma, 75 (8.7%, 75/858) were identified only in plasma (Table S8), yielding 48, 7, 5, and 18 patients harboring SNVs, InDels, fusions, and CNVs detected only in plasma. Lung squamous cell carcinoma (LUSC) and Lung adenocarcinoma (LUAD) are two major histological subtypes of NSCLC. Previous studies have indicated different performances of plasma testing in LUSC and LUAD. A concordance study was subsequently performed for LUSC and LUAD in the cohort. Our study revealed that the by‐variant sensitivity for targetable variants in ctDNA samples between LUAD and LUSC was comparable (70.2% vs. 58.3%, *p* = 0.038, Figure S3) when tissue‐NGS was used as a reference.

**TABLE 4:** The concordance between ctDNA and tumor tissue samples in detecting molecular variants.

variations	Total	Match (TP)	Tissue only (FN)	Plasma only (FP)	PPA	NPA	PPV
SNV	457	309	100	48	75.55%	99.70%	86.36%
InDel	284	218	59	7	78.70%	99.79%	96.89%
Fusion	109	76	28	5	73.08%	99.81%	93.83%
CNV	124	28	78	18	26.42%	98.94%	60.87%
Therapeutically targetable alterations							
*EGFR* G719X	11	7	4	0	63.64%	100%	100%
*EGFR* 19del	233	180	48	5	78.95%	99.26%	97.3%
*EGFR* L858R	193	141	41	11	77.47%	98.48%	92.76%
*EGFR* T790M	94	51	18	25	73.91%	97.01%	67.11%
*EGFR* S768I	5	4	1	0	80%	100%	100%
*EGFR* exon20 insertion	28	18	9	1	66.67%	99.89%	94.74%
*EGFR* L861Q	13	9	4	0	69.23%	100.00%	100%
*ALK* fusion (A19‐20)	75	52	21	2	71.23%	99.76%	96.3%
*ROS1* fusion (R31‐36)	18	14	2	2	87.5%	99.77%	87.5%
*RET* fusion (R10‐12)	16	10	5	1	66.67%	99.89%	90.91%
*MET* ex14 skipping mutation	7	5	1	1	83.33%	99.98%	83.33%
*NTRK* fusion	1	1	0	0	100%	100%	100%
*KRAS* G12X	69	53	15	1	77.94%	99.88%	98.15%
*BRAF* V600E	9	4	4	1	50%	99.89%	80%

*Note*: Positive concordance = (number of mutations positive in both tissue and ctDNA)/number of mutations positive in either tissue or ctDNA; A19‐20: fused to *ALK* exon 19–20; R31‐36: fused to *ROS1* exon 31–36; R10‐12: fused to *RET1* exon 10–12.

Abbreviations: CNV, copy number variant; ctDNA, circulating‐tumor DNA.*ERBB2* mutations include *ERBB2* exon 20 insertions and point mutation S310X; InDel, small insertion and indel; LGR, large genomic rearrangement; *MET* ex14 skipping, *MET* exon 14 skipping mutation; No, number; NSCLC, non‐small cell lung cancer; PPV, positive predictive value; SNV, single‐nucleotide variant.

**FIGURE 5:**
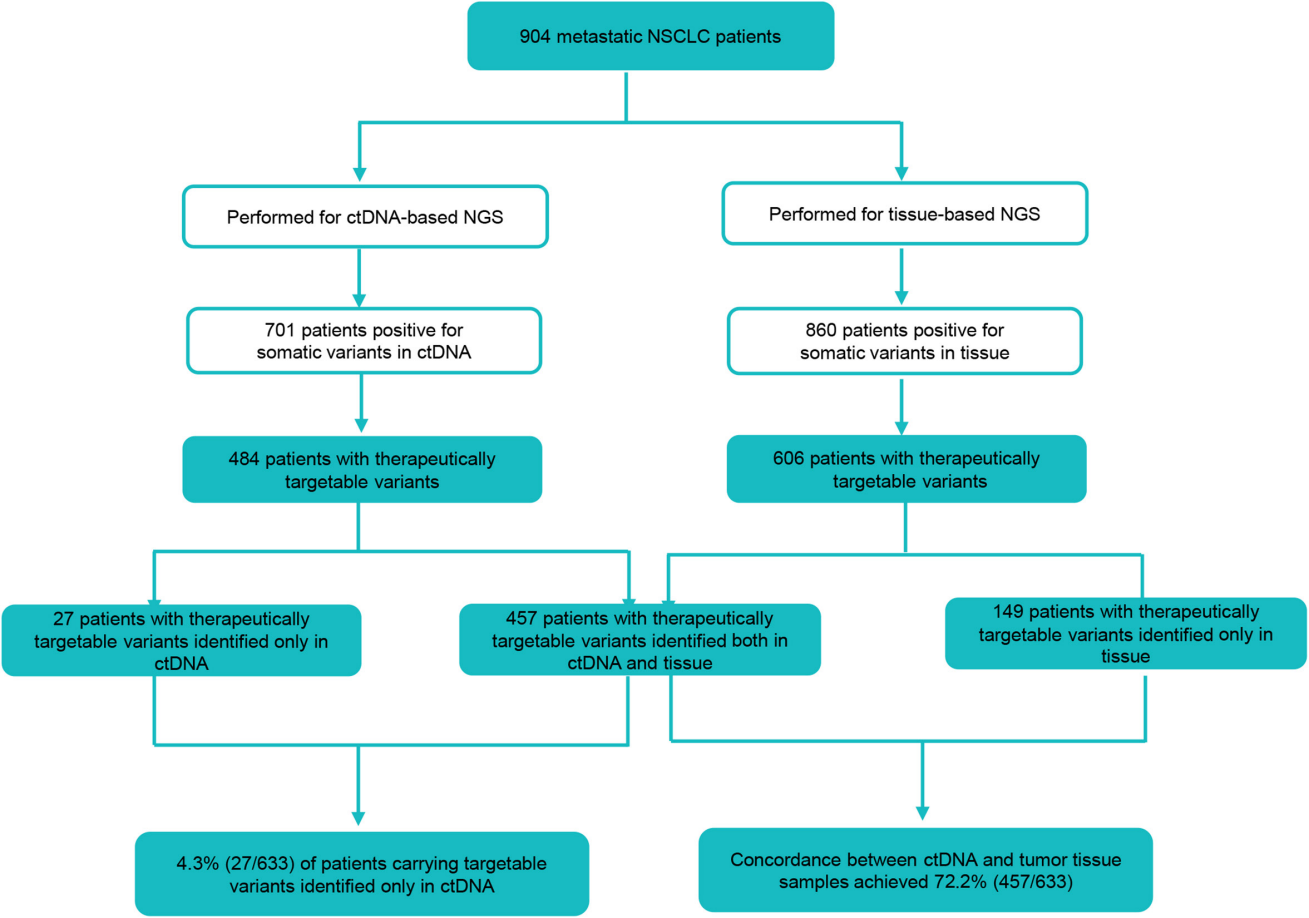
The performance of ctDNA in detecting targetable variants from metastatic NSCLC patients. ctDNA, circulating tumor DNA; NGS, next‐generation sequencing; NSCLC, non‐small cell lung cancer.

Collectively, of 904 patients performed for NGS, 633 patients harbored targetable variants, including 457 patients with shared targetable variants, 149 patients with targetable variants in tissue, and 27 patients (4.3%, 27/633) with targetable variants only in plasma, yielding a concordance of 72.2% (457/633) between ctDNA and tissue samples. Additionally, 4.3% (27/633) of patients carrying targetable variants were identified only in ctDNA (Figure 5). These findings suggest that 76.5% (484/633) of actionable variant‐driven NSCLC patients could be identified by upfront ctDNA NGS in clinical practice and that ctDNA testing is essential to complement DNA testing.

## 4 DISCUSSION

In this study, the basic properties of the 101‐test for detecting molecular variants, including LoB, LoD, sensitivity, specificity, and precision, were established by examining in silico simulated samples, commercial cfDNA reference standards, and in vitro serial dilution cell lines. In addition, the concordance between ctDNA and tumor tissue samples in detecting targetable variants between tumors was investigated in a large real‐world study of metastatic NSCLC patients.

The 101‐test showed a near‐zero false‐positive rate for panel‐wide variants. The results demonstrated the ultrahigh specificity of the 101‐test. LoD studies in commercial cfDNA reference standards and pooled cell line samples revealed the 101‐test could accurately detect hotspot SNVs, non‐hotspot SNVs, hotspot InDels, non‐hotspot InDels, and fusions down to an AF of 0.38%, 1%, 0.33%, 1%, and 0.33%, respectively, and detect CNV down to a CN of 2.5. These results suggest that the 101‐test had a higher sensitivity in the detection of hotspot SNVs/indels compared to non‐hotspot SNVs/indels. FoundationOne® Liquid CDx and Guardant360® CDx are two commercial liquid biopsy NGS companion diagnostic tests approved by the FDA to detect genomic variants in plasma.^22^, ^23^ The established LoD of Guardant360® CDx is as low as 0.2% for detecting *EGFR* exon 19 deletions/L858R/and T790M/exon 20 insertions, and 0.5% and 0.8% for detecting *KRAS* G12V and *NRAS* Q61R, respectively.^22^ The established LoD of Guardant360® CDx is down to a CN of 2.3 and 2.4 for identifying *ERBB2* and *MET* amplification, respectively.^22^ For the FoundationOne® Liquid CDx, the estimated LoD for short variants (substitutions and small InDels) was 0.40% for the enhanced sensitivity region and 0.82% for the standard sensitivity region of the bait set.^23^ Based on these data, we found that the performance of the 101‐test in detecting hotpot variants was comparable to that of the above two assays. Moreover, the 101 test used in the present study covers the most frequently mutated exons and introns of oncology‐related genes and has the potential to facilitate clinical practice due to its high‐efficiency library construction and low depth sequencing requirement. In the present study, instead of simply comparing NGS of ctDNA and tissue samples, we provided a thoroughly validation of the performance of the 101 test in ctDNA samples through in silico simulation experiments, LoD determination and precision studies in vitro experiments in reference cfDNA and cell lines, and clinical validation in paired plasma and tissue samples. These experiments thoroughly validated the performance of the 101‐gene test, not only providing ample evidence for the utility of the test in clinical practice but also offering guidance for the future development of similar panels within the field.

In our study, approximately 70% of the patients with metastatic NSCLC harbored at least one actionable alteration(s). *NTRK* fusions are rare in NSCLC, with an estimated frequency of 0.1% in NSCLC,^24^ which was similar to the 0.11% observed in our study. The prevalence of *EGFR* sensitizing and resistance variants, *ALK* fusion, *MET* exon 14 skipping mutation, *BRAF* V600E, *ROS1* fusion, *RET* fusion, and *ERBB2* variants in the present study were consistent with those in Chinese NSCLC patients reported in previous studies.^3^, ^25^, ^26^, ^27^ *MET* amplification had a frequency of 8.74% in our cohort, which is higher than the previously reported incidence of 3% in Asian patients.^28^, ^29^ It is well‐documented that *MET* amplification contributes to the resistance mechanism to EGFR‐TKI treatment in 5%–20% of advanced NSCLCs.^30^, ^31^ The higher frequency of *MET* amplification in our study is largely due to the inclusion of more patients who progressed to EGFR‐TKI treatment and underwent re‐biopsy.

Previous studies have demonstrated that the sensitivity of ctDNA for detecting targetable variants reached 60%–70% in advanced or metastatic NSCLC when tumor tissue was used as a reference standard.^18^, ^32^, ^33^ A similar result was observed in this large cohort study in the 101‐test showed a by‐patient sensitivity of 72.2%. Previous studies have demonstrated an unsatisfactory sensitivity of plasma testing for CNVs with a range of 3%–50%.^34^, ^35^, ^36^ Consistent with previous studies, the by‐variant sensitivity for CNVs (26.42%) was lower than that for SNVs (75.55%), indels (78.70%), or fusions (73.08%) in this study. More specifically, the by‐variant sensitivities for *MET* and *ERBB2* amplification were 23.5% and 31.6%, respectively. Previous studies and our study suggest that the detection of CNVs from plasma samples of patients with metastatic stage is challenging. The NSCLC NCCN guidelines (Version 3.2022) in 2022 recommend molecular testing for *EGFR* S768I and that *EGFR* S768I‐positive patients benefit from afatinib and osimertinib.^6^ The by‐variant sensitivity of *EGFR* S768I was 80.0%, with a PPV of 100%. Our work indicates the feasibility of ctDNA as an alternative to tumor tissue samples in predicting targetable SNVs/InDels/fusions.

In our study, the concordance rate between ctDNA and tissue samples in identifying targetable variants is 72.2%, which was aligned with previous large‐scale tissue‐plasma studies.^37^ Additionally, we found that 8.7% of targetable variants and 4.3% of patients harboring targetable variants were detected only in ctDNA. The discrepancy may be attributed to the spatial tumor heterogeneity.^37^ Biopsy tissue samples obtained from different parts of a tumor may harbor different variants, and ctDNA can overcome this heterogeneity.^37^ The present findings highlights the importance of ctDNA testing as a complementary approach to tissue testing.

A previous study documented that ctDNA might be a feasible biomarker in LUSC due to a higher ctDNA detection frequency in early stage LUSC than in early stage LUAD.^38^ Because patients with early stage NSCLC were not included, the comparison of ctDNA detection rates between LUSC and LUAD was not performed. Additionally, we observed a comparable by‐variant sensitivity in detecting targetable variants in metastatic LUSC and LUAD (58.3% and 70.2%, respectively). Our study suggests that ctDNA‐based NGS is an acceptable tool for detecting targetable variants in both LUAD and LUSC at the metastatic stage.

Our study has some limitations. A large, prospective study is needed to verify the associations between targetable variants and histological subtypes, and to verify the feasibility of ctDNA as an alternative source for detecting targetable variants in metastatic patients with insufficient tumor tissue specimens.

In conclusion, our findings demonstrated the feasibility and reliability of the 101‐test as a qualitative NGS‐based in vitro diagnostic method for detecting targetable variants. Our work also demonstrated the feasibility of using ctDNA as an alternative and/or complement to the tissue.

## AUTHOR CONTRIBUTIONS

**Feifei Fan:** Conceptualization (equal); writing – original draft (equal). **Guozhong Jiang:** Conceptualization (equal); writing – original draft (equal). **Juan Lv:** Conceptualization (equal); visualization (equal); writing – original draft (equal). **Hongmin Wang:** Data curation (equal). **Wenjie Li:** Visualization (equal); writing – original draft (equal). **Chenglin Liu:** Data curation (equal); formal analysis (equal). **Yu Zhao:** Data curation (equal); formal analysis (equal). **Zhou Zhang:** Software (equal); visualization (equal). **Haiwei Du:** Writing – original draft (equal). **Zhihong Zhang:** Conceptualization (equal); writing – review and editing (equal). **Xiangnan Li:** Conceptualization (equal); writing – review and editing (equal). **Wencai Li:** Conceptualization (equal); writing – review and editing (equal).

## FUNDING INFORMATION

This work was supported by a grant from the National Natural Science Foundation of China (No. 82172941 to Guozhong Jiang).

## CONFLICT OF INTEREST STATEMENT

The authors declare that they have no conflicts of interest.

## ETHICAL APPROVAL

This study was conducted in accordance with the Declaration of Helsinki and approved by the Institutional Review Board of the First Affiliated Hospital of Zhengzhou University.

## CONSENT TO PARTICIPATE

Informed consent was obtained from all subjects for the use of their plasma and tumor tissue samples.

## Supporting information


Figure S1:

Table S1:

## Data Availability

The data reported in this paper were deposited in the GVM,^39^, ^40^ China National Center for Bioinformation/Beijing Institute of Genomics, Chinese Academy of Sciences (https://ngdc.cncb.ac.cn/gvm/getProjectDetail?project=GVM000438).
