# Supplementary material for: Analytical and clinical validation of a NGS panel in detecting targetable variants from ctDNA of metastatic NSCLC patients
Source: Cancer Med. 2024 Oct 9;13(19):e70078. doi: 10.1002/cam4.70078 (PMC11464656; doi:10.1002/cam4.70078)
Supplement: Supplementary file 1 — Figure S1: [file CAM4-13-e70078-s002.docx]

**
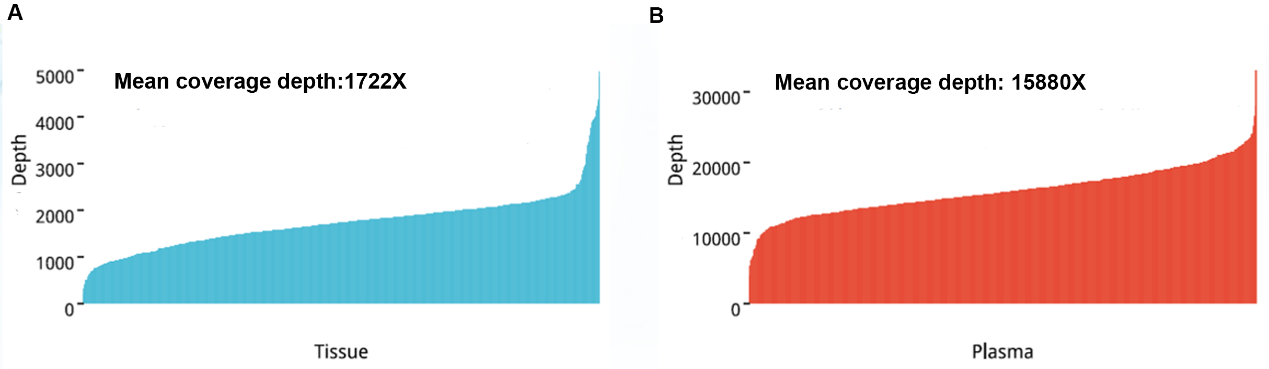
**

**Figure S1: The coverage depth for targeted sequencing for tissue samples (A) and ctDNA samples (B).**

**
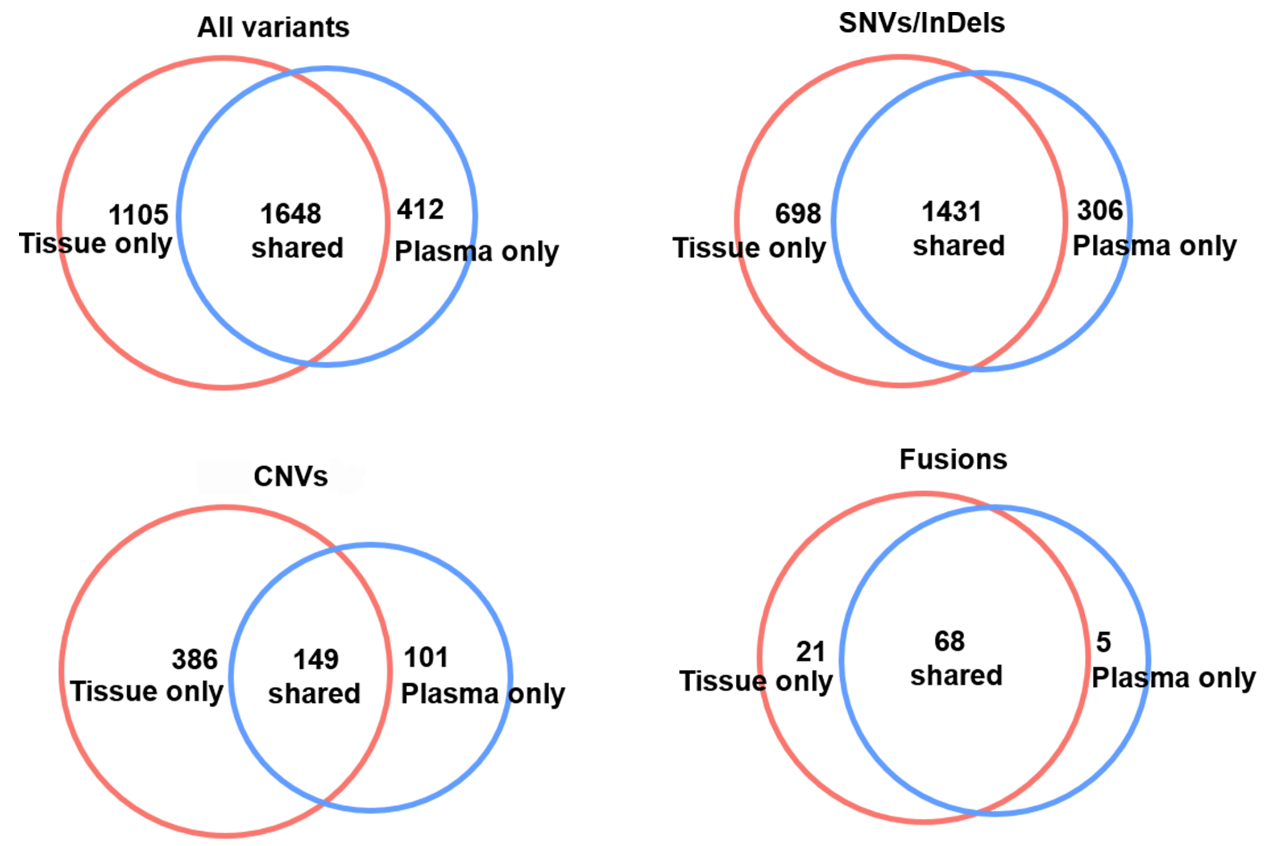
**

**Figure S2: The distribution of genomic alterations in tumor tissue and ctDNA samples.** A, the distribution of all genomic variants in tissue and ctDNA samples; B, the distribution of SNVs/InDels in tissue and ctDNA samples; C, the distribution of CNVs in tissue and cTDNA samples; D, the distribution of fusions in tissue and ctDNA samples. SNVs: single nucleotide variants; InDels: insertions and deletions; CNVs: copy number variants; ctDNA: circulating tumor DNA.


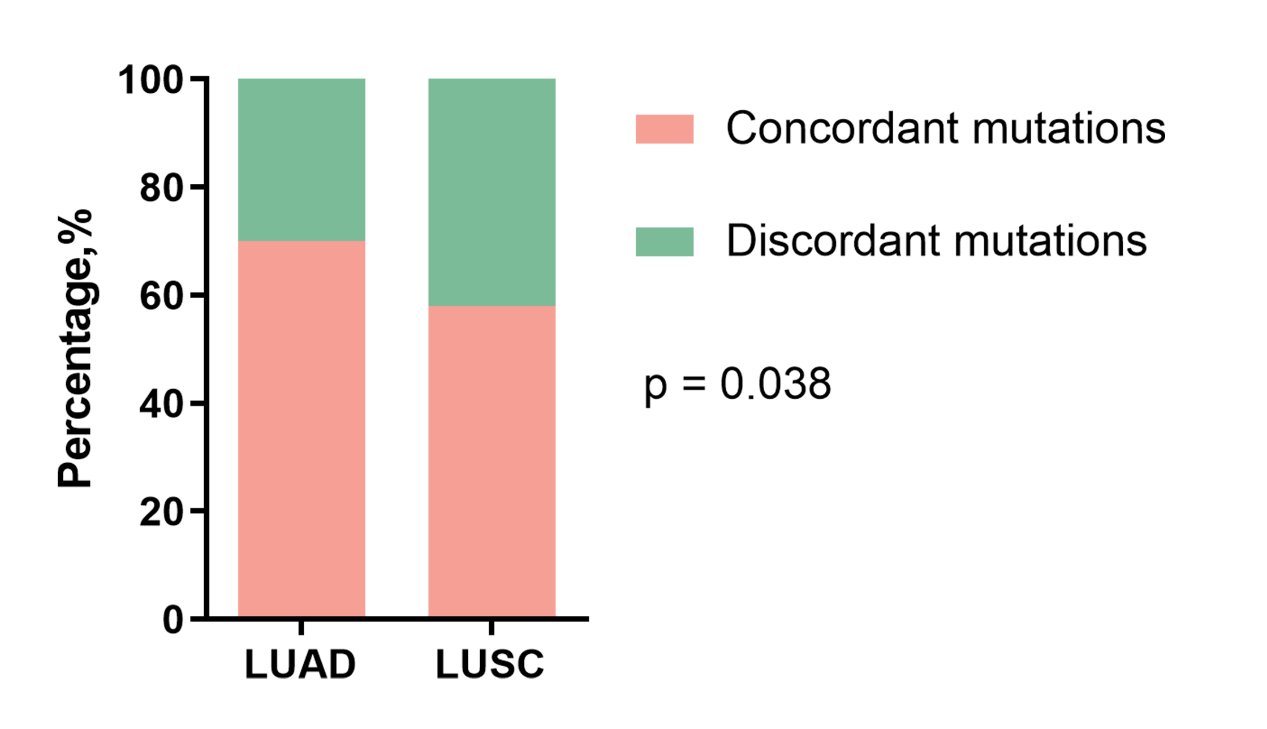


**Figure S3: The difference of by-variant sensitivity for targetable variants in ctDNA samples between LUAD and LUSC.** ctDNA: circulating tumor DNA; LUAD: lung adenocarcinoma; LUSC: lung squamous cell carcinoma.
