# Supplementary material for: Analytical and clinical validation of a NGS panel in detecting targetable variants from ctDNA of metastatic NSCLC patients
Source: Cancer Med. 2024 Oct 9;13(19):e70078. doi: 10.1002/cam4.70078 (PMC11464656; doi:10.1002/cam4.70078)
Supplement: Supplementary file 2 — Table S1: [file CAM4-13-e70078-s001.docx]

**Table S1 Gene list of the NGS panel**

| *AKT1* | *ALK* | *APC* | *AR* | *ARID1A* | *ATM* | *ATR* | *B2M* | *BARD1* | *BRAF* |
| --- | --- | --- | --- | --- | --- | --- | --- | --- | --- |
| *BRCA1* | *BRCA2* | *CCND1* | *CD274* | *CDK4* | *CDK6* | *CDKN1A* | *CDKN1B* | *CDKN2A* | *CHEK2* |
| *CTNNB1* | *EGFR* | *ERBB2* | *ERBB3* | *ERBB4* | *ESR1* | *FANCA* | *FANCI* | *FAT3* | *FBXW7* |
| *FGF19* | *FGF3* | *FGF4* | *FGFR1* | *FGFR2* | *FGFR3* | *FLT1* | *FLT3* | *FLT4* | *GATA3* |
| *HRAS* | *IDH1* | *IDH2* | *JAK1* | *JAK2* | *KDR* | *KEAP1* | *KIT* | *KMT2D* | *KRAS* |
| *MAP2K1* | *MET* | *MLH1* | *MRE11* | *MSH2* | *MSH6* | *MTOR* | *MYC* | *MYCN* | *NBN* |
| *NF1* | *NFE2L2* | *NOTCH1* | *NRAS* | *NTRK1* | *NTRK2* | *NTRK3* | *PALB2* | *PDGFRA* | *PDGFRB* |
| *PIK3CA* | *PIK3CG* | *PIK3R1* | *PMS2* | *POLD1* | *POLE* | *PTEN* | *RAD50* | *RAD51B* | *RAD51C* |
| *RAD51D* | *RAD54L* | *RAF1* | *RARA* | *RB1* | *RET* | *RNF43* | *ROS1* | *SMAD4* | *SMARCA4* |
| *STK11* | *TERT* | *TP53* | *TP63* | *VEGFA* | *VEGFB* | *VEGFC* | *VHL* | *YES1* | *CD74* |
| *SDC4* |  |  |  |  |  |  |  |  |  |

NGS: next-generation sequencing.

**Table S2 Details of variants evaluated in pools of genomic DNA from human cancer cell lines**

| **Cancer cell lines** | **cell line mix** | **Mutation counts** | | | | |
| --- | --- | --- | --- | --- | --- | --- |
|  |  | **AF:0.125-0.25%** | **AF:**  **0.25-0.5%** | **AF:**  **0.5-1%** | **AF:**  **>1%** | **Total** |
| NCI-H596, HCC78,  NCI-H2122, KARPAS299, LC-2/ad,  MDA-MB468, SW948,  CL11,  NCI-H1975, NCI-H1650, BT20,  SW48,  NB-4,  A-549, RPMI8226, HCC1599, Jurkat,  C32,  HCC1937 | Pooled DNA sample 1-1 | 3 | 4 | 5 | 103 | 115 |
|  | Pooled DNA sample 1-2 | 4 | 3 | 23 | 82 | 112 |
|  | Pooled DNA sample 1-3 | 4 | 21 | 56 | 27 | 108 |
|  | Pooled DNA sample 1-4 | 22 | 55 | 23 | 3 | 103 |
|  | Pooled DNA sample 1-5 | 51 | 23 | 3 | 0 | 77 |
| NCI-H3122, H2228,  HCT-15, HCC1954, Hs746T,  PSN-1,  K562,  A2058,  H441,  SW1573, HCC827,  HCC-1143, PANC-1,  SK-BR3 | Pooled DNA sample-2-1 | 4 | 6 | 0 | 77 | 87 |
|  | Pooled DNA sample-2-2 | 6 | 0 | 12 | 66 | 84 |
|  | Pooled DNA sample-2-3 | 0 | 12 | 42 | 24 | 78 |
|  | Pooled DNA sample-2-4 | 10 | 43 | 15 | 7 | 75 |
|  | Pooled DNA sample-2-5 | 44 | 15 | 4 | 3 | 66 |

AF: allele frequency.

**Table S3 Details of variants evaluated in in silico samples**

| **SNV/InDel** | |
| --- | --- |
| Hotspot (49) | SNV (37) |
|  | MNV (3) |
|  | Insertion (5) |
|  | Deletion (4) |
| Non-hotspot (73) | SNV (20) |
|  | MNV (2) |
|  | Insertion (22) |
|  | Deletion (29) |
| **Fusion** | |
| *ALK* | *EML4*-*ALK* (E21:A20) |
| *RET* | *CCDC6*-*RET* (C1:R12) |
| *FGFR1* | *BAG4*-*FGFR1* (B2:F6) |
| *FGFR2* | *KIAA1598*-*FGFR2* (K6:F18) |
| *FGFR3* | *FGFR3*-*TACC3* (F17:T11) |
| *ROS1* | *CD74*-*ROS1* (C7:R33) |
|  | *SDC4*-*ROS1* (S4:R33) |
| *NRG1* | *CD74*-*NRG1* (C7:N2) |
| *NTRK1* | *MPRIP*-*NTRK1* (M21:N14) |
| *NTRK3* | *ETV6*-*NTRK3* (E4:N13) |

SNV: single-nucleotide variant; MNV: multi-nucleotide variant.

**Table S4 Analytical validation of molecular variants using *in silico* data**

| **Mutation Type** | **Expected AF** | **Replicates** | **Unique mutation** | **Total Mutation** | **TP** | **FN** | **FP** | **Sensitivity** | **PPV** |
| --- | --- | --- | --- | --- | --- | --- | --- | --- | --- |
| **Hotspot**  **SNVs** | 0.1% | 60 | 40 | 2400 | 2027 | 373 | 0 | 84.46% | 100% |
|  | 0.3% | 60 | 40 | 2400 | 2400 | 0 | 0 | 100% | 100% |
|  | 0.5% | 60 | 40 | 2400 | 2399 | 1 | 0 | 99.96% | 100% |
|  | 0.7% | 60 | 40 | 2400 | 2400 | 0 | 0 | 100% | 100% |
|  | 1% | 60 | 40 | 2400 | 2400 | 0 | 0 | 100% | 100% |
|  | 2% | 60 | 40 | 2400 | 2400 | 0 | 0 | 100% | 100% |
| **Non-hotspot SNV** | 0.1% | 60 | 22 | 1320 | 0 | 1320 | 0 | 0.00% | NA |
|  | 0.3% | 60 | 22 | 1320 | 0 | 1320 | 0 | 0.00% | 100% |
|  | 0.5% | 60 | 22 | 1320 | 1068 | 252 | 0 | 80.90% | 100% |
|  | 0.7% | 60 | 22 | 1320 | 1273 | 47 | 0 | 96.44% | 100% |
|  | 1% | 60 | 22 | 1320 | 1320 | 0 | 0 | 100% | 100% |
|  | 2% | 60 | 22 | 1320 | 1320 | 0 | 0 | 100% | 100% |
| **Hotspot**  **InDels** | 0.1% | 60 | 9 | 540 | 359 | 201 | 0 | 64.11% | 100% |
|  | 0.3% | 60 | 9 | 540 | 536 | 4 | 0 | 99.26% | 100% |
|  | 0.5% | 60 | 9 | 540 | 537 | 3 | 0 | 99.44% | 100% |
|  | 0.7% | 60 | 9 | 540 | 540 | 0 | 0 | 100% | 100% |
|  | 1% | 60 | 9 | 540 | 540 | 0 | 0 | 100% | 100% |
|  | 2% | 60 | 9 | 540 | 540 | 0 | 0 | 100% | 100% |
| **Non-hotspot**  **InDels** | 0.1% | 60 | 51 | 3060 | 177 | 2883 | 0 | 5.78% | NA |
|  | 0.3% | 60 | 51 | 3060 | 251 | 2809 | 0 | 8.24% | 100% |
|  | 0.5% | 60 | 51 | 3060 | 2478 | 582 | 0 | 80.98% | 100% |
|  | 0.7% | 60 | 51 | 3060 | 2997 | 63 | 0 | 97.94% | 100% |
|  | 1% | 60 | 51 | 3060 | 3034 | 26 | 0 | 99.15% | 100% |
|  | 2% | 60 | 51 | 3060 | 3051 | 9 | 0 | 99.71% | 100% |
| **Fusions** | 0.2% | 60 | 9 | 540 | 511 | 31 | 0 | 94.63% | 100% |
|  | 0.4% | 60 | 9 | 540 | 530 | 10 | 0 | 98.15% | 100% |
|  | 0.6% | 60 | 9 | 540 | 534 | 6 | 0 | 98.89% | 100% |
|  | 0.8% | 60 | 9 | 540 | 537 | 3 | 0 | 99.44% | 100% |
|  | 1% | 60 | 9 | 540 | 536 | 4 | 0 | 99.26% | 100% |
| **CNVs** | 2.1 | 260 | 13 | 260 | 6 | 254 | 0 | 2.31% | 100% |
|  | 2.3 | 260 | 13 | 260 | 241 | 19 | 0 | 92.69% | 100% |
|  | 2.5 | 260 | 13 | 260 | 260 | 0 | 0 | 100% | 100% |
|  | 2.7 | 260 | 13 | 260 | 259 | 1 | 0 | 99.62% | 100% |

AF: mutant allele frequency; SNV: single-nucleotide variant; InDel: insertion and deletion; CNV: copy number variant; PPV: positive predictive value; TP: true positive; FN: false negative; FP: false positive; NA: not available.

**Table S5 Details of variants evaluated in commercial cfDNA reference**

| **Gene** | **Mutation** | **Mutation Type** |
| --- | --- | --- |
| *AKT1* | p.E17K | SNV (Hotspot) |
| *ALK* | TPR-ALK(T15:A20) | Fusion |
| *APC* | p.R1450* | SNV (Non-hotspot) |
| *APC* | p.T1556Nfs*3 | Insertion (Non-hotspot) |
| *BRAF* | p.V600E | SNV (Hotspot) |
| *CTNNB1* | p.T41A | SNV (Non-hotspot) |
| *EGFR* | p.E746_A750delELREA | Deletion (Hotspot) |
| *EGFR* | p.D770_N771insG | Insertion (Hotspot) |
| *EGFR* | p.L858R | SNV (Hotspot) |
| *EGFR* | p.T790M | SNV (Hotspot) |
| *ERBB2* | p.Y772_A775dupYVMA | Insertion (Hotspot) |
| *FGFR3* | p.S249C | SNV (Non-hotspot) |
| *FLT3* | p.D835Y | SNV (Non-hotspot) |
| *IDH1* | p.R132C | SNV (Non-hotspot) |
| *JAK2* | p.V617F | SNV (Hotspot) |
| *KIT* | p.D816V | SNV (Hotspot) |
| *KRAS* | p.G12D | SNV (Hotspot) |
| *NRAS* | p.Q61R | SNV (Hotspot) |
| *PDGFRA* | p.S566Qfs*6 | Insertion (Non-hotspot) |
| *PIK3CA* | p.*1069Mfs*4 | Insertion (Non-hotspot) |
| *PIK3CA* | p.E545K | SNV (Hotspot) |
| *PIK3CA* | p.H1047R | SNV (Hotspot) |
| *PTEN* | p.K267Rfs*9 | Deletion (Non-hotspot) |
| *PTEN* | p.P248Tfs*5 | Insertion (Non-hotspot) |
| *RET* | p.M918T | SNV(Non-hotspot) |
| *RET* | NCOA4-RET(N8:R12) | Fusion |
| *SMAD4* | p.A466Gfs*28 | Insertion (Non-hotspot) |
| *TP53* | p.C242Afs*5 | Deletion (Non-hotspot) |
| *TP53* | p.R175H | SNV (Hotspot) |
| *TP53* | p.R248Q | SNV (Hotspot) |
| *TP53* | p.R273H | SNV (Hotspot) |
| *TP53* | p.S90Pfs*33 | Deletion (Non-hotspot) |

cfDNA: cell-free DNA; SNV: single-nucleotide variant.

**Table S6 Baseline characteristics of Chinese patients with metastatic NSCLC**

| **Characteristics** | **n (%)** |
| --- | --- |
| **Median age (range), years** | 61 (28-94) |
| **Gender** |  |
| Female | 406 (44.9%) |
| Male | 498 (55.1%) |
| **Histology** |  |
| LUAD | 842 (93.1%) |
| LUSC | 62 (6.9%) |
| **Brain metastasis** |  |
| With | 189 (20.9%) |
| Without | 607 (67.1%) |
| Unknown | 108 (12.0%) |
| **Smoking history** |  |
| With | 151 (16.7%) |
| Without | 450 (49.8%) |
| Unknown | 303 (33.5%) |
| **Chemotherapy history** |  |
| With | 242 (26.8%) |
| Without | 586 (64.8%) |
| Unknown | 76 (8.4%) |
| **TKI treatment history** |  |
| With | 272 (30.1%) |
| Without | 603 (66.7%) |
| Unknown | 29 (3.2%) |
| **Total** | **904** |

NSCLC: non-small cell lung cancer; LUAD: lung adenocarcinoma; LUSC: lung squamous cell carcinoma; TKI: tyrosine kinase inhibitor.

**Table S7 Prevalence of targetable variants in ctDNA and tumor tissue samples**

|  | **No. of patients detected in ctDNA samples** | **Prevalence in ctDNA** | **No. of patients detected in tissue samples** | **Prevalence in tissue samples** | **No. of patients detected in tissue or ctDNA samples** | **Prevalence in tissue or ctDNA samples** |
| --- | --- | --- | --- | --- | --- | --- |
| **Targetable variants** | 484 | 53.54% | 606 | 67.04% | 628 | 69.47% |
| ***EGFR* mutation** | 376 | 41.59% | 465 | 51.44% | 480 | 53.1% |
| ***ALK* fusion** | 44 | 4.87% | 59 | 6.53% | 60 | 6.64% |
| ***MET* amplification** | 27 | 2.99% | 68 | 7.52% | 79 | 8.74% |
| ***MET* exon 14 skipping** | 6 | 0.66% | 6 | 0.66% | 7 | 0.77% |
| ***ERBB2* amplification** | 19 | 2.1% | 38 | 4.2% | 45 | 4.98% |
| ***ERBB2* 20ins/S310X** | 24 | 2.65% | 25 | 2.77% | 26 | 2.88% |
| ***BRAF* V600E** | 5 | 0.55% | 8 | 0.88% | 9 | 1% |
| ***ROS1* fusion** | 14 | 1.55% | 14 | 1.55% | 16 | 1.77% |
| ***RET* fusion** | 11 | 1.22% | 13 | 1.44% | 14 | 1.55% |
| ***NTRK* fusion** | 1 | 0.11% | 1 | 0.11% | 1 | 0.11% |

ctDNA: circulating tumor DNA; *EGFR* mutations including *EGFR* sensitizing and resistance mutations; *ERBB2* mutations including exon 12 insertion and point mutation S310X; *EGFR*: epidermal growth factor receptor; *BRAF*: B-Raf proto-oncogene, serine/threonine kinase; *ERBB2*: erb-b2 receptor tyrosine kinase 2; *ALK*: ALK (anaplastic lymphoma kinase) receptor tyrosine kinase; *RET*: ret proto-oncogene; *ROS1*, ROS proto-oncogene 1, receptor tyrosine kinase; *MET*: MET (hepatocyte growth factor receptor gene) proto-oncogene, receptor tyrosine kinase; *NTRK*: neurotrophic tyrosine kinase receptor; No.: number.

**Table S8 Concordance analysis for variants between ctDNA and tumor tissue samples in NSCLC patients**

| **Mutation type** | **Total** | **No. of concordant alterations** | **Tissue only** | **Plasma only** | **Sensitivity** | **PPV** | **Positive concordance, %** |
| --- | --- | --- | --- | --- | --- | --- | --- |
| **All variants** | 3165 | 1648 | 1105 | 412 | 59.9% | 80.0% | 52.1% |
| SNV/InDel | 2435 | 1431 | 698 | 306 | 67.2% | 82.4% | 68.8% |
| CNV | 636 | 149 | 386 | 101 | 34.3% | 59.6% | 23.4% |
| Fusion | 94 | 68 | 21 | 5 | 76.4% | 93.1% | 72.3% |
| **Targetable variants** | 858 | 548 | 235 | 75 | 70% | 88.0% | 63.9% |
| *EGFR* sensitizing/resistance mutation | 602 | 423 | 129 | 50 | 76.6% | 89.4% | 70.3% |
| *ALK* fusion | 60 | 43 | 16 | 1 | 72.9% | 97.7% | 71.7% |
| *BRAF* V600E | 9 | 4 | 4 | 1 | 50% | 80% | 44.4% |
| *ROS1* fusion | 16 | 12 | 2 | 2 | 85.7% | 85.7% | 75.0% |
| *RET* fusion | 14 | 10 | 3 | 1 | 76.9% | 90.9% | 71.4% |
| *MET* amplification | 79 | 16 | 52 | 11 | 23.5% | 59.3% | 20.3% |
| *MET* exon 14 skipping mutations | 7 | 5 | 1 | 1 | 83.3% | 83.3% | 71.4% |
| *ERBB2* amplification | 45 | 12 | 26 | 7 | 31.6% | 63.2% | 26.7% |
| *ERBB2* 20ins/S310X | 26 | 23 | 2 | 1 | 92% | 95.8% | 88.5% |

NSCLC: non-small cell lung cancer; SNV: single-nucleotide variant; InDel: small insertion and indel; CNV: copy number variant; No.: number; PPV: positive predictive value. *MET* ex14 skipping: *MET* exon 14 skipping mutation; *ERBB2* mutations including *ERBB2* exon 20 insertions and point mutation S310X; ctDNA: circulating-tumor DNA. Positive concordance = (number of mutations positive in both tissue and cfDNA)/number of mutations positive in either tissue or cfDNA or in both
